# Nomogram for predicting the unfavourable outcomes of percutaneous endoscopic transforaminal discectomy for lumbar disc herniation: a retrospective study

**DOI:** 10.3389/fsurg.2023.1188517

**Published:** 2023-06-02

**Authors:** Xiaofeng Jiang, Lili Gu, Gang Xu, Xuezhong Cao, Jian Jiang, Daying Zhang, Mu Xu, Yi Yan

**Affiliations:** ^1^ Department of Radiology, The First Affiliated Hospital of Nanchang University, Nanchang, China; ^2^ Institute of Pain Medicine, Jiangxi Academy of Clinical and Medical Sciences, Nanchang, China; ^3^ Department of Pain Medicine, The First Affiliated Hospital of Nanchang University, Nanchang, China

**Keywords:** percutaneous endoscopic transforaminal discectomy, lumbar disc herniation, nomogram, prediction model, lower back pain

## Abstract

**Objective:**

To investigate and integrate multiple independent risk factors to establish a nomogram for predicting the unfavourable outcomes of percutaneous endoscopic transforaminal discectomy (PETD) for lumbar disc herniation (LDH).

**Methods:**

From January 2018 to December 2019, a total of 425 patients with LDH undergoing PETD were included in this retrospective study. All patients were divided into the development and validation cohort at a ratio of 4:1. Univariate and multivariate logistic regression analyses were used to investigate the independent risk factors associated with the clinical outcomes of PETD for LDH in the development cohort, and a prediction model (nomogram) was established to predict the unfavourable outcomes of PETD for LDH. In the validation cohort, the nomogram was validated by the concordance index (C-index), calibration curve, and decision curve analysis (DCA).

**Results:**

29 of 340 patients showed unfavourable outcomes in the development cohort, and 7 of 85 patients showed unfavourable outcomes in the validation cohort. Body mass index (BMI), course of disease (COD), protrusion calcification (PC), and preoperative lumbar epidural steroid injection (LI) were independent risk factors associated with the unfavourable outcomes of PETD for LDH and were identified as predictors for the nomogram. The nomogram was validated by the validation cohort and showed high consistency (C-index = 0.674), good calibration and high clinical value.

**Conclusions:**

The nomogram based on patients' preoperative clinical characteristics, including BMI, COD, LI and PC, can be used to accurately predict the unfavourable outcomes of PETD for LDH.

## 1. Introduction

Low back pain is a leading cause of disability and absenteeism in the developed and developing countries (1). It not only reduces the quality of life for patients, but also causes an enormous economic burden to both health-care and social support systems (2, 3). Previous studies have shown that approximately 70% of people have low back pain in their lifetime, and with an annual prevalence of 15%–45% (4, 5). Up to half of the low back pain is caused by lumbar disc herniation (LDH), and the incidence of LDH has risen steeply and gradually affected more younger individuals in the past two decades (6).

At present, percutaneous endoscopic transforaminal discectomy (PETD) has been widely used in LDH due to the merits of normal paraspinal structures preservation, less soft tissue injury, fewer complications and shorter operation time (7). Most patients can relieve pain and return to normal life after PETD (8), but some patients have unfavourable outcomes postoperatively, and even need reoperation (9, 10). To improve clinical decision making and patient satisfaction, it is important to figure out the risk factors and predict the unfavourable outcomes of PETD for LDH.

Previous studies have identified several clinical factors as the significant risk factors associated with the clinical outcomes of PETD for LDH (11, 12), but they did not perform comprehensive analysis of these risk factors, which provided little help in improving clinical decision making. The development of nomograms make up for this deficiency, as nomograms can integrate a variety of significant risk factors and generate a single numerical estimation of event probability, which can be applied to predict the clinical outcomes of surgery (13). To our knowledge, only one study has developed a nomogram for pain and functional outcomes after lumbar spine fusion surgery (14), and this predictive model is rarely used in predicting the unfavourable outcomes of PETD for LDH.

Therefore, we performed a retrospective study to develop and validate a nomogram to predict the unfavourable outcomes of PETD for LDH.

## 2. Methods

### 2.1. Study design and patients

**Figure 1:**
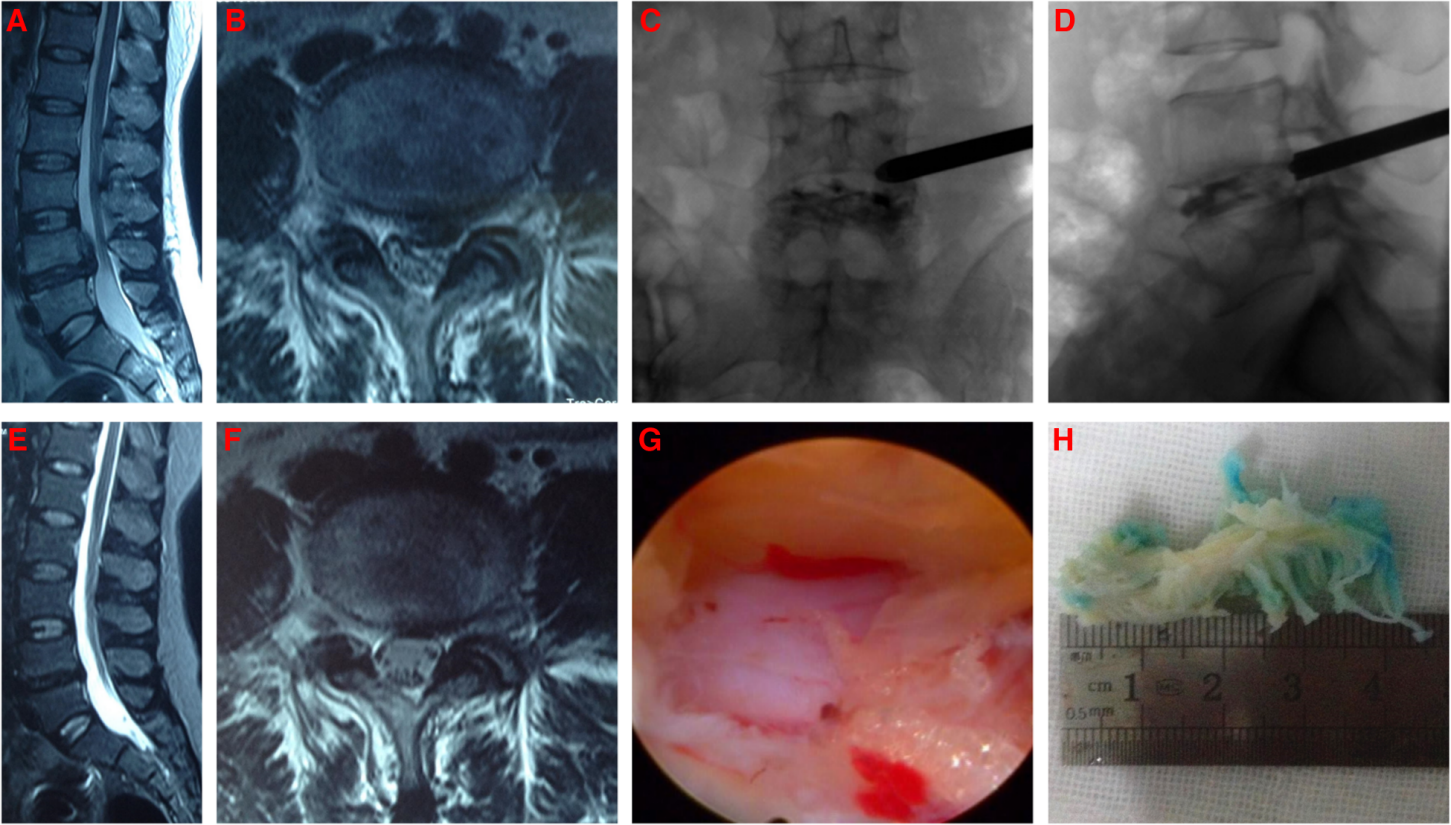
The typical PETD procedure. (**A,B**) Preoperative MRI of L4/5 LDH; (**C,D**) The working catheter of PETD; (**E,F**) 10 months postoperative MRI of L4/5 showing good decompression of nerve root; (**G**) Decompression of nerve root under endoscope; (**H**) Intraoperative removal of the nucleus pulposus.

From January 2018 to December 2019, LDH patients treated with PETD at the Department of Pain Medicine of The First Affiliated Hospital of Nanchang University were collected. The PETD was performed by two senior and experienced surgeons and the detailed surgical procedure was same as that described in our previous study (15) and was shown in Figure 1. The inclusion criteria were leg pain or leg pain + low back pain, diagnosis as single-segment LDH by computed tomography (CT) and/or magnetic resonance imaging (MRI), failure of conservative treatment for more than 2 months. The exclusion criteria were unclear diagnosis, multi-segment LDH, recurrent LDH, prior spine surgery, other lumbar diseases (tuberculosis, infection, tumour, etc), non-transforaminal approach or failure of the transforaminal approach and loss to follow-up. All patients were followed up for 12 months by outpatient or telephone and clinical outcomes were evaluated by the Numeric Rating Scale (NRS) and the modified MacNab criteria (16). The modified MacNab criteria evaluated as excellent or good and NRS < 3 were defined as favourable outcomes, and those evaluated as moderate or poor and NRS ≥ 3 were defined as unfavourable outcomes. A total of 447 patients with LDH who were treated with PETD were collected. Finally, 425 patients completed 12 months of follow-up and were included in this retrospective study. All patients were randomly divided in a ratio of 4:1 into the development and validation cohort by computer-generated random order (https://www.randomizer.org).

### 2.2. Variables for analysis

Clinical characteristics including age, gender, body mass index (BMI), history of lumbar trauma (LT), preoperative lumbar epidural steroid injection (LI), course of disease (COD), symptoms, segments, disc degeneration (DD), Modic change (MC) and protrusion calcification (PC) were collected from all patients. Age, gender, BMI, LT, COD, LI, symptoms and other clinical data were obtained from medical records or radiological examinations. Age was divided into ≥50 years and <50 years (17). BMI was classified as overweight (BMI ≥ 25 kg/m^2^) and normal (BMI < 25 kg/m^2^) according to World Health Organization standards; COD was divided into ≥6 months and <6 months (18). Symptoms were divided into leg pain and leg pain + low back pain. MC was assessed by MRI (19). DD was classified as mild (Pfirrmann grade Ⅰ-Ⅲ) or severe (Pfirrmann grade Ⅳ-Ⅴ) (20). PC was assessed by CT.

### 2.3. Statistical analysis

All the variables were categorical. Univariate and multivariate logistic regression analyses were used to investigate the independent risk factors associated with the clinical outcomes of PETD for LDH. The odds ratio (OR) and 95% confidence interval (CI) were analyzed by SPSS 22.0 software (SPSS Inc, Chicago, IL, USA). Then we put the data into R software (http://www.R-project.org) to establish the nomogram. The discrimination and calibration of the nomogram were validated by the calibration curve, concordance index (C-index) and decision curve analysis (DCA). The calibration curve, C-index and DCA curve were calculated and drawn by R software. *P* < 0.05 was considered statistically significant.

## 3. Results

### 3.1. Patient characteristics

**Figure 2:**
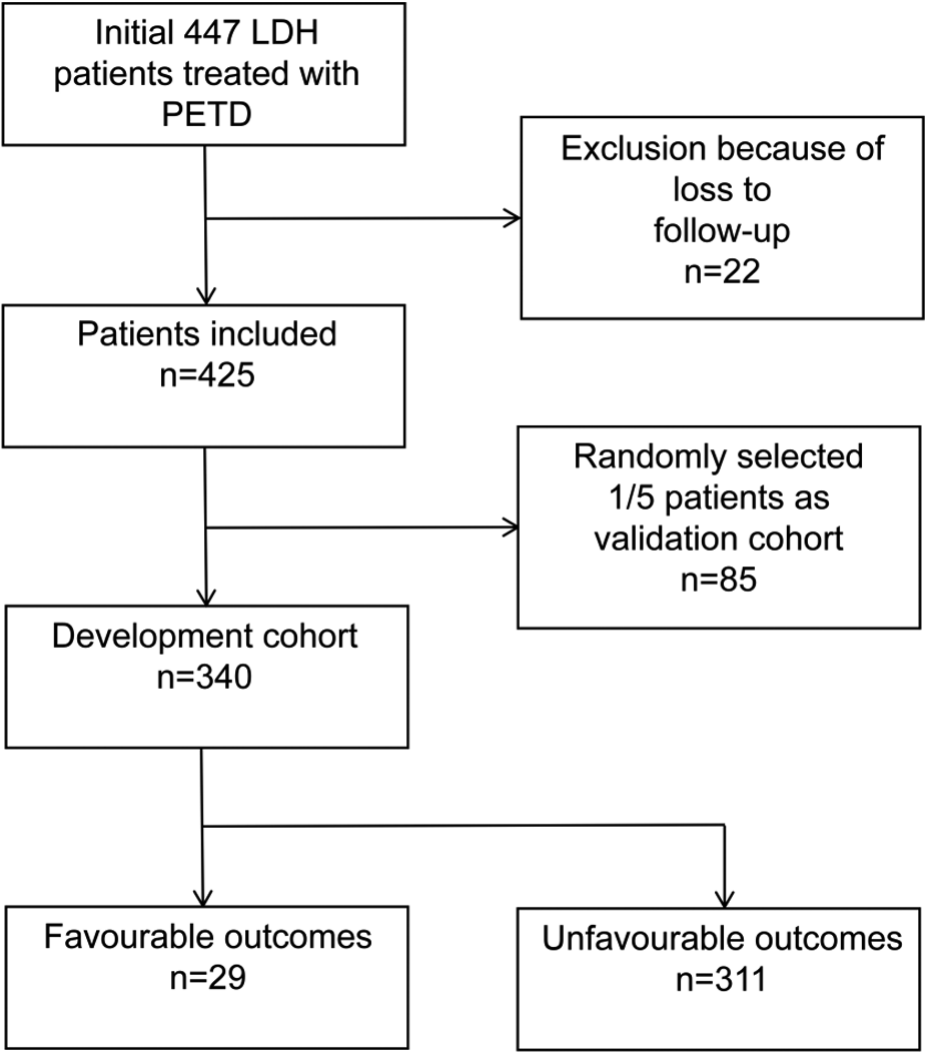
Flow chart of patients collection.

A total of 447 patients with LDH who were treated with PETD were collected. Finally, 425 patients completed 12 months of follow-up, and all patients were randomly divided in a ratio of 4:1 into the development cohort (*n* = 340) and the validation cohort (*n* = 85). Unfavourable outcomes of PETD for LDH were found in 29 of 340 patients in the development cohort, and 7 of 85 patients in the validation cohort (Figure 2). The clinic characteristics of the development and validation cohort were summarized in Table 1.

**Table 1:** Baseline clinic characteristics of development and validation cohort.

Characteristic	All cohort (*n *= 425, %)	Development cohort (*n *= 340, %)		Validation cohort (*n *= 85, %)	
		Favourable (*n *= 311, %)	Unfavourable (*n *= 29, %)	Favourable (*n *= 78, %)	Unfavourable (*n *= 7, %)
Gender					
Male	257 (60.5%)	185 (59.5%)	19 (65.5%)	51 (65.4%)	2 (28.6%)
Female	168 (39.5%)	126 (40.5%)	10 (34.5%)	27 (34.6%)	5 (71.4%)
Age (years)					
<50	320 (75.3%)	245 (78.8%)	12 (41.4%)	60 (76.9%)	3 (42.9%)
≥50	105 (24.7%)	66 (21.2%)	17 (58.6%)	18 (23.1%)	4 (57.1%)
BMI (Kg/m^2^)					
<25	323 (76.0%)	243 (78.1%)	14 (48.3%)	63 (80.8%)	3 (42.9%)
≥25	102 (24.0%)	68 (21.9%)	15 (51.7%)	15 (19.2%)	4 (57.1%)
LT					
No	400 (94.1%)	296 (95.2%)	27 (93.1%)	72 (92.3%)	5 (71.4%)
Yes	25 (5.9%)	15 (4.8%)	2 (6.9%)	6 (7.7%)	2 (28.6%)
COD (months)					
<6	160 (37.6%)	116 (37.3%)	4 (13.8%)	38 (48.7%)	2 (28.6%)
≥6	265 (62.4%)	195 (62.7%)	25 (86.2%)	40 (51.3%)	5 (71.4%)
Symptoms					
Leg pain	60 (14.1%)	46 (14.8%)	3 (10.3%)	11 (14.1%)	0 (0.0%)
Lower back pain + leg pain	365 (85.9%)	265 (85.2%)	26 (89.7%)	67 (85.9%)	7 (100.0%)
Segments					
L3/4	20 (4.7%)	8 (2.6%)	1 (3.4%)	11 (14.1%)	0 (0.0%)
L4/5	280 (65.9%)	205 (65.9%)	26 (89.7%)	46 (59.0%)	3 (42.9%)
L5/S1	125 (29.4%)	98 (31.5%)	2 (6.9%)	21 (26.9%)	4 (57.1%)
DD					
Mild	205 (48.2%)	161 (51.8%)	6 (20.7%)	38 (48.7%)	0 (0.0%)
Severe	220 (51.8%)	150 (48.2%)	23 (79.3%)	40 (51.3%)	7 (100.0%)
MC					
No	345 (81.2%)	259 (83.3%)	19 (65.5%)	63 (80.8%)	4 (57.1%)
Yes	80 (18.9%)	52 (16.7%)	10 (34.5%)	15 (19.2%)	3 (42.9%)
PC					
No	342 (80.5%)	264 (84.9%)	12 (41.4%)	64 (82.1%)	2 (28.6%)
Yes	83 (19.5%)	47 (15.1%)	17 (58.6%)	14 (17.9%)	5 (71.4%)
LI					
No	362(85.2%)	282(90.7%)	9(31.0%)	69(88.5%)	2(28.6%)
Yes	63(14.8%)	29(9.3%)	20(69.0%)	9(11.5%)	5(71.4%)

BMI, body mass index; LT, history of lumbar trauma; COD, course of disease; DD, disc degeneration; MC, Modic change; PC, protrusion calcification; LI, preoperative lumbar epidural steroid injection.

### 3.2. Univariate and multivariate logistic analyses in the development cohort

The univariate logistic analysis showed significant differences in age, BMI, COD, DD, MC, PC and LI (Table 2). Multivariate logistic regression analysis demonstrated that BMI, COD, LI and PC were independent risk factors associated with the unfavourable outcomes of PETD for LDH (Table 3).

**Table 2:** Univariate logistic regression analysis in the development cohort.

Characteristic	OR (95% CI)	*P*-value
Gender		
Male	Reference	0.756
Female	1.123 (0.539–2.342)	
Age (years)		
<50	Reference	<0.001
≥50	4.589 (2.170–9.703)	
BMI (Kg/m^2^)		
<25	Reference	<0.001
≥25	4.625 (2.187–9.781)	
LT		
No	Reference	0.986
Yes	1.014 (0.225–4.566)	
COD (months)		
<6	Reference	0.024
≥6	2.881 (1.152–7.204)	
Symptoms		
Leg pain	Reference	0.560
Lower back pain + leg pain	1.443 (0.420–4.957)	
Segments		
L3/4	Reference	0.182
L4/5	2.352 (0.301–18.355)	0.415
L5/S1	0.989 (0.109–8.977)	0.992
DD		
Mild	Reference	0.001
Severe	4.506 (1.804–11.253)	
MC		
No	Reference	<0.001
Yes	4.685 (2.352–9.546)	
PC		
No	Reference	<0.001
Yes	14.191 (6.183–32.573)	
LI		
No	Reference	<0.001
Yes	4.343 (2.249–8.386)	

BMI, body mass index; LT, history of lumbar trauma; COD, course of disease; DD, disc degeneration; MC, Modic change; PC, protrusion calcification; LI, preoperative lumbar epidural steroid injection.

**Table 3:** Multivariate logistic regression analysis in the development cohort.

Characteristic	OR (95% CI)	*P*-value
BMI (Kg/m^2^)		
<25	Reference	<0.001
≥25	7.417 (2.502–21.987)	
COD (months)		
<6	Reference	0.006
≥6	6.084 (1.674–22.107)	
PC		
No	Reference	<0.001
Yes	27.803 (8.295–93.189)	
LI		
No	Reference	<0.001
Yes	28.100 (8.620–91.600)	

BMI, body mass index; COD, course of disease; PC, protrusion calcification; LI, preoperative lumbar epidural steroid injection.

### 3.3. Establishment of the nomogram

**Figure 3:**
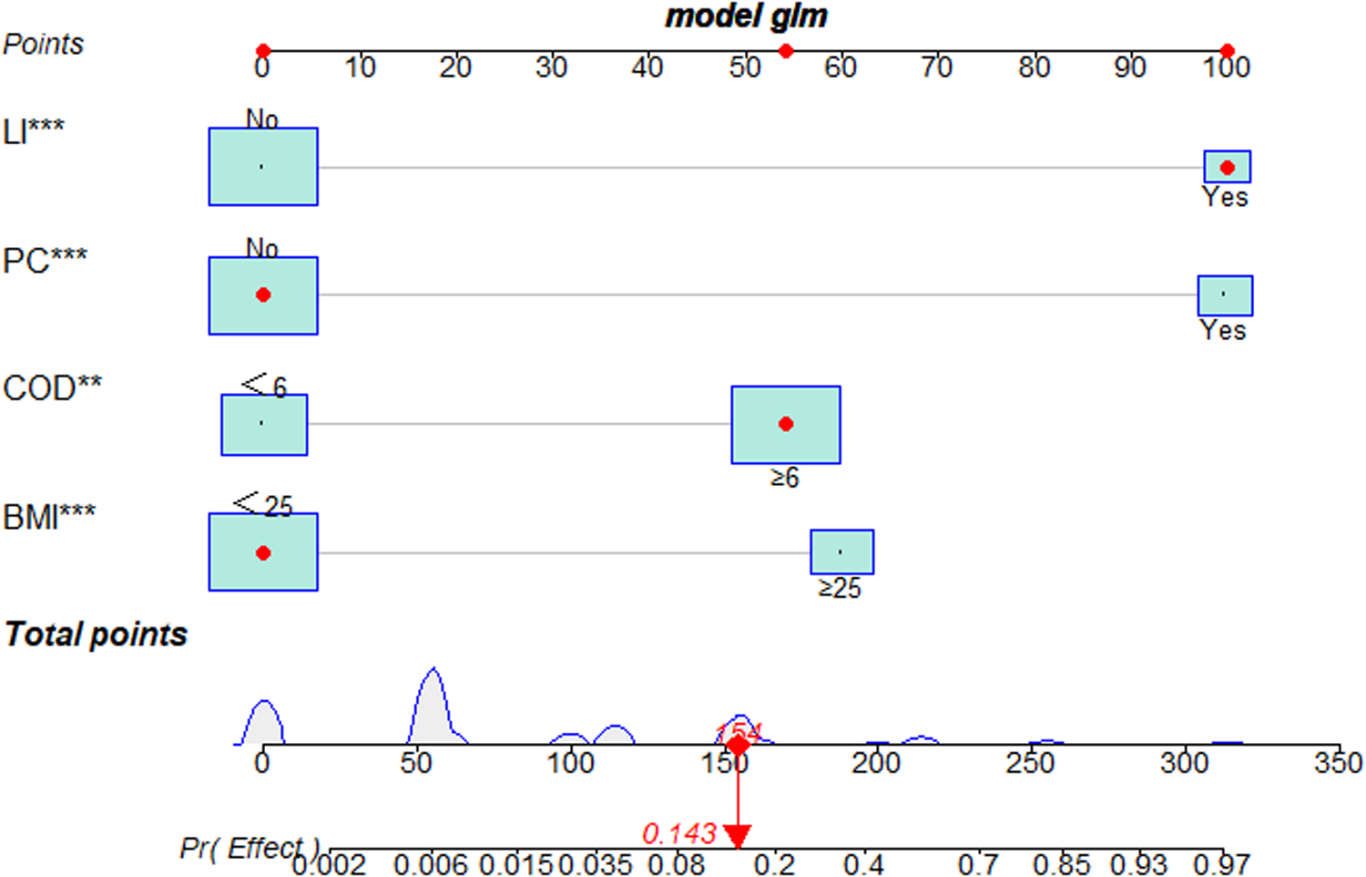
The nomogram for predicting the clinical outcomes of PETD for LDH. A representative of the LDH patient to show how to excute the nomogram. Each independent risk factor of the nomogram was scored on its corresponding points scale. The scores for four independent risk factors were then added to obtain the total score. A vertical line corresponded to the total score and generated a single numerical estimation of event probability, which can predict the unfavourable outcomes of PETD for LDH. The patient's total score was 154, which corresponds to a probability of 0.143 for having unfavorable outcomes of PETD for LDH (**: *P* < 0.01, ***:P < 0.001).

Univariate and multivariate logistic regression analyses showed that BMI, COD, LI and PC were independent risk factors associated with the clinical outcomes of PETD for LDH. Subsequently, these four independent risk factors were used to establish a nomogram. Each independent risk factor in the nomogram is scored based on its corresponding points scale. The scores for the four independent risk factors are added to obtain the total score. Finally, a vertical line is drawn down from the total score row to generate a single numerical estimation of the event probability, which can predict the unfavourable outcomes of PETD for LDH (Figure 3).

### 3.4. Validation of the nomogram

**Figure 4:**
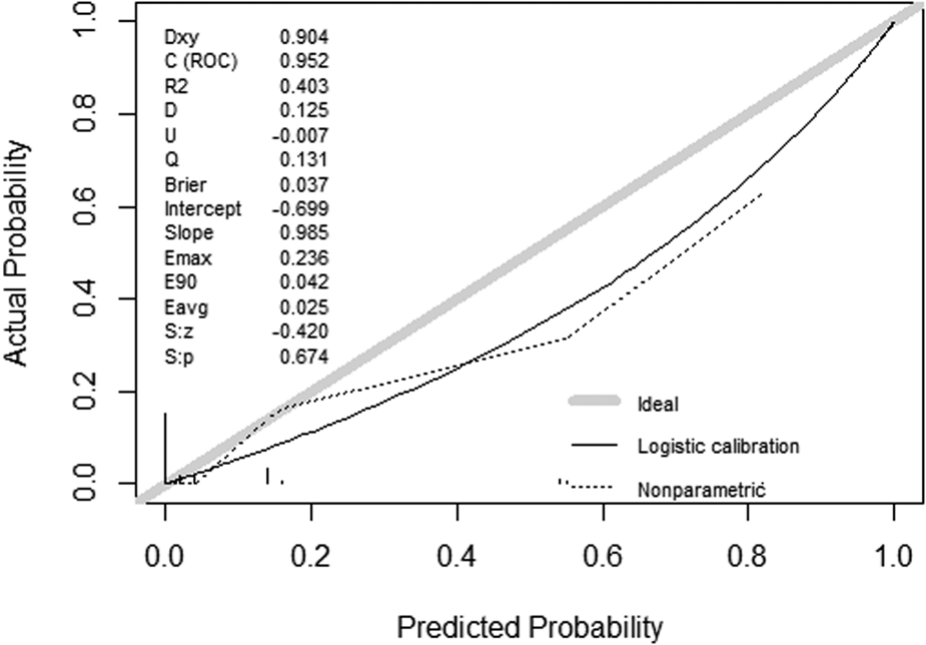
Calibration and discrimination of the nomogram. In the validation cohort, the prediction model showed good discrimination (C-index = 0.674) in differentiating the clinical outcomes of PETD for LDH, and the actual probability of the clinical outcomes of PETD for LDH was consistent with the probability predicted by the nomogram.

**Figure 5:**
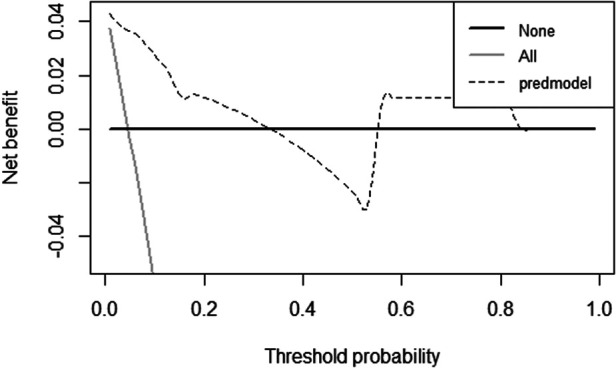
The decision curve analysis (DCA) curve for the clinical values of the nomogram. The DCA curve showed that the clinical value of the nomogram presented more net benefits at the threshold probability of 0%–32% and 58%–85%, indicating that the nomogram had good clinical efficacy.

In the validation cohort, the nomogram showed good discrimination (C-index = 0.837) in distinguishing the clinical outcomes of PETD for LDH. Moreover, we calculated the calibration curve, which showed that the regression fitting curve was close to the standard curve (*P* = 0.674) (Figure 4), meaning that the actual outcomes of PETD for LDH were consistent with the outcomes predicted by the nomogram. In addition, the DCA curve was conducted to calculate the clinical value of the nomogram by quantifying the net benefits at different threshold probability. The DCA curve showed that the clinical value of the nomogram presented more net benefits at the threshold probability of 0%–32% and 58%–85%, indicating that the nomogram had good clinical efficacy (Figure 5).

## 4. Discussion

The clinical outcomes of PETD for LDH can be influenced by multiple factors. Nonetheless, previous studies mainly focused on single risk factors that may influence the outcomes of PETD for LDH (11, 12), which did not contribute much to improve clinical decision making or patient satisfaction. Clinically, since few surgeons will act on the reason of a single risk factor, recent studies have focused on integrating multiple risk factors into a tool that can help guide clinical decision making (21). In this study, a prediction model (nomogram) was established for the first time to predict the unfavourable outcomes of PETD for LDH by integrating multiple independent risk factors to provide reliable evidence for clinical decision making.

Similar to previous studies, the present study revealed that COD (18) and BMI (22) were independent risk factors associated with the clinical outcomes of PETD for LDH. In the early stage of LDH, the nucleus pulposus does not adhere with the intraspinal tissues, which can be completely removed by forceps (as shown in Figure 1H). However, a long course of disease might lead to epidural venous congestion or epidural adhesions and even protrusion calcification, which increase the surgical difficulty and result in nucleus pulposus residue and even surgical failure. Jeffrey et al. (18) showed that LDH patients with a course of disease longer than 6 months had a longer operative time and increased intraoperative bleeding. Moreover, our previous study found that chronic low back pain can affect brain structure and function, leading to neuropathic pain (23). Heuch et al. (24) suggested that low back pain was associated with increased levels of BMI. Böstman (25) revealed that the BMI of LDH patients undergoing surgery was 25.1–27.3 kg/m^2^, while 22.3–23.1 kg/m^2^ in the general population, indicating that obese patients are more likely to have LDH. In addition, obese patients who undergo surgery for LDH have a longer operative time, more intraoperative bleeding and longer hospital stays (22). Consistent with previous correlational study (26), the results of this study suggested that BMI was an independent risk factor associated with the clinical outcomes of PETD for LDH, and LDH patients with BMI ≥ 25 kg/m^2^ were more likely to have unfavourable outcomes after PETD. High BMI may influence the biomechanical characteristics of discs, especially in degenerated and postoperative discs (26). Therefore, obese patients should prolong the time of using the lumbar belt and gradually reduce their weight after PETD. If obese patients can not reduce the load of discs, there is still a risk of recurrent LDH or LDH in other lumbar segments (26). For this reason, Meredith et al. (27) suggested that weight loss counseling should be considered in the preoperative conversation.

LI is one of the most commonly non-surgical treatments of LDH between drugs and surgery (28). The North American Spine Society has recommended that LI for LDH as a grade A choice. LI in the treatment of LDH has been shown to be effective, where with a wide variation in reported efficacy (29). For the varational efficacy, we think that LI can eliminate inflammation, relieve neuroedema, and has a good effect on early mild LDH (30), which can successfully prevent surgical intervention (31). However, for severe chronic LDH, LI could not remove the nucleus pulposus and may result in poor outcomes. Consistent with our results, Koltsov et al. (32) revealed that patients with preoperative LI did experience higher rates of reoperation than those with no preoperative LI. Additionally, Bhattacharjee et al. (33) believed that steroid may impede annulus fibrosus healing and thus predispose poor outcomes after surgery. PC is more common in patients with a longer course of disease (34). Previous study has reported that PC is associated with chronic inflammation and immunity (35). Due to the limitation of the endoscopic visual field, it is difficult to completely remove PC, and with high risk of nerve root injury to expose the PC fragment (36). Further, partial removal of PC may affect the stability of the posterior tissues of the intervertebral disc, and form new fissures and tears postoperatively, which leads to recurrent LDH. Moreover, the removal of PC requires a dynamic bur, which greatly damages the normal structure of the spinal canal and may cause epidural adhesion and fibrosis, leading to postoperative pain (37). We believe that the key to PETD for LDH is the decompression of the nerve root. If PC is directly related to nerve root compression, it should be removed as much as possible; if not, it should be retained to enhance the stability of the posterior tissues of the intervertebral disc and prevent recurrent LDH.

Pathologically, the four independent risk factors associated with the clinical outcomes of PETD for LDH are closely related. With the developed nomogram, we can perform a comprehensive analysis of these independent risk factors. Nomograms are visual format of the predictive model that allow improved predictive accuracy for outcomes by calculating the cumulative effect of each independent risk factor compared with the previous studies. Thus, surgeons can preoperatively predict the expected clinical outcomes of PETD for LDH based on the patients' preoperative clinical characteristics. In the validation cohort, the prediction model showed good discrimination (C-index = 0.674) in differentiating the clinical outcomes of PETD for LDH, and the actual probability of the clinical outcomes of PETD for LDH was consistent with the probability predicted by the nomogram. In terms of clinical efficacy, the prediction model showed good clinical efficacy, compared with the extreme curves in the threshold probability of 0%-32% and 58%-85%, indicating that the prediction model had high clinical efficacy and safety.

There are some limitations in this retrospective study. Firstly, this study established a prediction model for the clinical outcomes of PETD for LDH based on patients’ preoperative clinical characteristics but did not include related intraoperative and postoperative risk factors, such as surgeon experience (38), lifestyle and inappropriate physical workload (39). However, based on the results of this study, surgeons can improve clinical decision making, predict the expected postoperative clinical outcomes and guide postoperative rehabilitation. Secondly, we used a validation cohort independent of the development cohort to validate the prediction model and avoid overfitting. However, if the nomogram is validated with data from other hospitals, the results would be more convincing. Finally, this study is a single-centre, retrospective study, may have potential biases in patient collection. A multi-centre study with a larger sample size and more related risk factors may further optimise and validate the model and confirm its value in clinical practice.

## 5. Conclusions

The prediction model (nomogram) based on patients' preoperative clinical characteristics, including BMI, COD, LI and PC, can be used to accurately predict the unfavourable outcomes of PETD for LDH. As a reliable, simplified and well-understood scoring system, the nomogram can be implemented in clinical practice to aid surgeons in clinical decision making, and it can allow for a more informed and well-understood discussion with patients regarding the expected clinical outcomes when considering PETD for LDH.

## Data Availability

The raw data supporting the conclusions of this article will be made available by the authors, without undue reservation.
